# Changes in the Medical Corps of the United States Army

**Published:** 1918-11

**Authors:** 


### WAR MEDICINE

Published by the American Red Cross Society in France
for the
Medical Officers of the American Expeditionary Forces

**EDITORIAL OFFICES:** 2, Place de Rivoli, Paris, Rooms 422-425

WAR MEDICINE *accepts no original articles. Its scope is limited to the publication of the proceedings of the Research Society of the American Red Cross in France, abstracts of original articles, and editorial comment on subjects pertaining to the medicine, surgery, and hygiene of the war.*

*Circulars, bulletins, and reports from the Office of the Chief Surgeon of the American Expeditionary Forces will also appear in* WAR MEDICINE.

WAR MEDICINE *is distributed free of charge to the medical officers of the American Expeditionary Forces.*

*All communications pertaining to the mailing list should be addressed to the Editorial Offices. If copies are not received promptly and regularly the managing editor should be informed at once. Addresses should be written distinctly.*

THE RESEARCH SOCIETY OF THE AMERICAN RED CROSS IN FRANCE

*All communications should be addressed to the Secretary,*
*2, Place de Rivoli, Paris, Room 422*

### EDITORIAL COMMENTS

### CHANGES IN THE MEDICAL CORPS OF THE UNITED STATES ARMY

Major-General W. C. Gorgas, having reached, on his 64th birthday, the age of enforced legal retirement in the United States Army, has been relieved of his duties as Surgeon-General. There have been many expressions of regret at the retirement of General Gorgas. He is one of the most beloved men in American medicine, and is recognized everywhere as being the greatest sanitarian that the world has ever produced. Certainly no medical officer in the United States Army has ever had throughout his career a record of greater usefulness or achievement. Announcement has not yet been made as to the future activities of General Gorgas, but the talents of this great sanitarian will surely be utilized in a large way in some capacity. Newspaper reports state that he will work in sanitation under the Rockefeller International Health Commission, and there has also been a rumor that he will be in governmental public health work in the United States. It is known that General Gorgas dreams of eradicating yellow fever from the world, and, by the sanitation of one or two ports on the coast of South America, as was done with Havana and the Panama, the first infectious disease will have been stamped out of existence. Whatever General Gorgas may do, it is the wish of everyone that he may have many years of continued usefulness and happiness ahead of him.

### Surgeon-General Ireland.

The selection of Major-General W. M. Ireland to succeed General Gorgas as Surgeon-General is a deserved promotion for one of the most efficient general officers in the history of the United States Army. In organizing and building up the Medical Department of the American Expeditionary Forces to its present high state of efficiency, General Ireland has shown that he is a man of broad vision and of great executive ability. He carries into the Surgeon-General's Office a long experience in various administrative capacities. For a number of years he was Aide to Surgeon-General O'Reilley, and therefore knows thoroughly the details of the Surgeon-General's Office. He has seen service also in the Philippines and on the Mexican border, and his experience in France, added to his other qualifications for the position, makes him the logical man to correlate and co-ordinate the work of the Surgeon-General's Office in Washington with that of the Medical Department of the American Expeditionary Forces. The universal popularity of General Ireland with the medical officers of the Regular Medical Corps and the Medical Reserve Corps is a tribute to his fairness and sound judgment. He also has the absolute confidence of the general staff of the Expeditionary Forces, and has the respect and esteem of every one with whom he has been associated. All who known General Ireland predict for him a record of great achievement as Surgeon-General of the United States Army.

### Chief Surgeon W. D. McCaw.

In the appointment of Colonel W. D. McCaw as Chief Surgeon of the American Expeditionary Forces, General Pershing has promoted a man who has filled with distinction a number of the most important positions in the Medical Corps of the United States Army, and who, by training and ability, is thoroughly fitted for the high position to which he has been called. Colonel McCaw served in the Spanish-American War in Cuba. He was later Chief Surgeon in the Philippines, and was Chief Surgeon with the Army of mobilization with General Pershing in the Southern Department on the Mexican border. General Pershing, therefore, knows personally the efficiency of the officer whom he is placing in charge of the Medical Department of the American Expeditionary Forces. Colonel McCaw has seen service not only in the field, but he has had long experience in administrative and executive work, having been in the Surgeon-General's Office in Washington for a number of years. Since Colonel McCaw has been in France, he has been the head of the Sanitary Department, and has been associated with General Ireland in the Chief-Surgeon's Office. He is, therefore, thoroughly familiar with the duties of the position which he now occupies, and there can be no question of the continued efficiency of the Chief Surgeon's Office.

### Other Changes.

Brigadier-General J. R. Kean, who has been the Executive Officer in the Chief-Surgeon's Office in the American Expeditionary Forces, has gone to Washington with General Ireland to take up his duties in that capacity in the Surgeon-General's Office.

Brigadier-General F. A. Winter, who has been Chief Surgeon of the Medical Department of the American Expeditionary Forces in England, has been detailed to serve as Executive Officer in the Chief Surgeon's Office in France.

Colonel H. A. Shaw, formerly Chief Surgeon of Base

Section No. 2, has been detailed to the Chief Surgeon's Office in charge of the Division of Sanitation.

Colonel Bailey K. Ashford has gone to Washington, having been assigned to the general staff of the United States Army. He therefore leaves the Field Service Sanitary School which he established, and of which he was the Commanding Officer. Lieutenant-Colonel R. C. MacDonald has been made Director, and has been placed in charge of the Field Service Sanitary School.

### PROMOTIONS.

Colonel James D. Glennan, in charge of the Hospitalization in the Chief Surgeon's Office, has been promoted to the grade of Brigadier-General.

Colonel J. M. T. Finney, Chief Consultant in Surgery, and Colonel W. S. Thayer, Chief Consultant in Medicine, are the first Medical Reserve Corps Officers to receive the rank of Brigadier-General.

The long-delayed promotions of Medical Reserve Corps Officers from the grade of Colonel down to Captain, which the Medical Department of the American Expeditionary Forces has been endeavouring to secure for some time, are now being rapidly made. Congratulations to many deserving medical officers are therefore in order.

